# P-1506. *In Vitro* Activity of Ceftazidime-Avibactam and Comparator Agents against *Pseudomonas aeruginosa* Collected from Patients with Presumed Hospital- and Community-acquired Respiratory Tract Infections as a Part of the ATLAS Global Surveillance Program 2018-2022

**DOI:** 10.1093/ofid/ofae631.1675

**Published:** 2025-01-29

**Authors:** Henry Li, Mark Estabrook, Gregory Stone, Daniel F Sahm

**Affiliations:** IHMA, Schaumburg, Illinois; IHMA, Schaumburg, Illinois; Pfizer, Inc., Groton, Connecticut; IHMA, Schaumburg, Illinois

## Abstract

**Background:**

The β-lactamase inhibitor, avibactam, has potent inhibitory activity against Class A, Class C, and some Class D serine β-lactamases. This study evaluated the *in vitro* activity of ceftazidime-avibactam and comparators against respiratory tract infection (RTI)-associated *Pseudomonas aeruginosa* isolated from presumed community-acquired (CA; culture started < 48 hours after hospital admission) and hospital-acquired (HA; culture started ≥48 hours post-admission) infections collected from 2018-2022.

**Figure:**
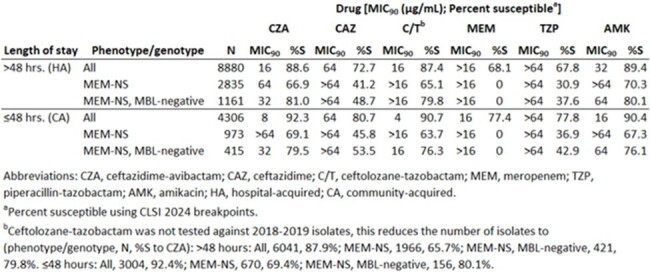

**Methods:**

13,186 isolates were collected from the lower respiratory tract of patients from 224 medical centers in 57 countries (excluding China and North America). Of these, 8,880 were HA and 4,306 were CA. MICs were determined using CLSI broth microdilution and interpreted using CLSI 2024 breakpoints. Isolates testing nonsusceptible (NS) to meropenem (MEM) were screened by PCR and Sanger sequencing for β-lactamase genes.

**Results:**

Ceftazidime-avibactam was the most active agent tested against the CA population (92.3% susceptible, MIC_90_ of 8 µg/mL; Table). Against HA isolates, ceftazidime-avibactam was active against 88.6% (MIC_90_ of 16 µg/mL). Only amikacin was active against more HA isolates (89.4% susceptible; MIC_90_ 32 µg/mL). Of isolates that were MEM-NS and metallo-β-lactamase (MBL)-negative, 81.0% of HA isolates and 79.5% of the CA isolates were susceptible.

**Conclusion:**

The *in vitro* activity of ceftazidime-avibactam against CA and HA organisms infecting the lower respiratory tract indicates that it remains an important option for treating infections including those caused by MEM-NS organisms that do not carry an MBL.

**Disclosures:**

**Henry Li, MS in Biotechnology**, Pfizer, Inc.: Advisor/Consultant **Mark Estabrook, MS**, Pfizer, Inc.: Advisor/Consultant **Daniel F. Sahm, PhD**, Pfizer, Inc.: Advisor/Consultant

